# Effect of In Ovo Injection of L-Arginine in Different Chicken Embryonic Development Stages on Post-Hatchability, Immune Response, and Myo-D and Myogenin Proteins

**DOI:** 10.3390/ani9060357

**Published:** 2019-06-14

**Authors:** Sivakumar Allur Subramaniyan, Da Rae Kang, Jin Ryong Park, Sharif Hasan Siddiqui, Palanisamy Ravichandiran, Dong Jin Yoo, Chong Sam Na, Kwan Seob Shim

**Affiliations:** 1 Department of Animal Biotechnology, College of Agriculture and Life Sciences, Chonbuk National University, Jeonju 561756, Korea; sivaphdbio@gmail.com (S.A.S.); kangdr92@gmail.com (D.R.K.); wlsfyd1321@naver.com (J.R.P.); hasanshuhin@gmail.com (S.H.S.); 2 Department of Energy Storage/Conversion Engineering, Graduate School, Hydrogen and Fuel Cell Research Center, Chonbuk National University, Jeonju 561756, Korea; 3 Department of Life Science, Chonbuk National University, Jeonju 561756, Korea; ravichandru55@gmail.com (P.R.); djyoo@jbnu.ac.kr (D.J.Y.)

**Keywords:** embryonic development, heat shock proteins, immunoglobulin, intracytoplasmic vacuoles, L-arginine

## Abstract

**Simple Summary:**

In the current study, we hypothesized that the in ovo injection of L-arginine (L-Arg) at different stages of embryonic development, which would have positive effects on the survival rate, hatching rate, immunoglobulin M (IgM) levels, heat shock proteins (HSPs) such as HSP-47, HSP-60, and HSP-70, and muscle development markers as well: Mainly, myoblast determination protein (myoD) and myogenin in pectoral muscles. As indicated, the in ovo injection of L-Arg resulted in an increased hatch rate and weight, survival rate, higher levels of IgM, and myogenin and MyoD expression in the muscles. At the same time, a decrease in the level of HSP-47, HSP-60, and HSP-70 expressions in the tissues was observed on the 14th day of injection compared to the eighth and 18th day of the injection period. In addition, the in ovo injection of L-Arg decreased the serum glutamate oxaloacetate transaminase (SGOT) and serum glutamate pyruvate transaminase (SGPT) concentration in serum as well micronuclei and nuclear abnormality in the blood on the 14th day of the incubation period. Hence, the 14th day would be a suitable day for the injection of L-Arg to promote the hatching rate and muscle growth of broiler chickens.

**Abstract:**

The aim of this study was to evaluate the effect of in ovo injection with different ratios of L-arginine (L-Arg) into Ross broiler eggs at three different embryonic developmental stages (eighth day (d), 14th day, and 18th day) on the survival, hatchability, and body weight (BW) of one-day-old hatched chicks. Additionally, we have analyzed the levels of serum glutamate oxaloacetate transaminase (SGOT) and serum glutamate pyruvate transaminase (SGPT), the protein expression of heat shock proteins (HSPs), and we have also determined micronuclei (MN) and nuclear abnormality (NA). In addition, the genotoxic effect was observed in peripheral blood cells such as the presence of micronuclei and nuclear abnormalities in the experimental groups. The results showed that survival and hatching rates as well as body weight were increased on the 14th day of incubation compared to the eighth and 18th day of incubation at lower concentrations of L-Arg. Moreover, the levels of SGOT and SGPT were also significantly (*p* < 0.05) increased on the 14th day of incubation at the same concentration (100 μg/μL/egg) of injection. In addition, immunoglobulin (IgM) levels were increased on the 14th day of incubation compared to other days. The protein expressions of HSP-47, HSP-60, and HSP-70 in the liver were significantly down-regulated, whereas the expression of myogenin and myoblast determination protein (MyoD) were significantly up-regulated on the 14th day after incubation when treated with all different doses such as 100 μg, 1000 μg, and 2500 μg/μL/egg, namely 3T1, 3T2, and 3T3, respectively. However, the treatment with low doses of L-Arg down-regulated the expression levels of those proteins on the 14th day of incubation. Histopathology of the liver by hematoxylin and eosin (H&E) staining showed that the majority of liver damage, specifically intracytoplasmic vacuoles, were observed in the 3T1, 3T2, and 3T3 groups. The minimum dose of 100 μg/mL/egg on the 14th day of incubation significantly prevented intracytoplasmic vacuole damages. These results demonstrate that in ovo administration of L-Arg at (100 μg/μL/egg) may be an effective method to increase chick BW, hatch rate, muscle growth-related proteins, and promote the immune response through increasing IgM on the 14th day of the incubation period.

## 1. Introduction

The selection of chickens (*Gallus gallus*) for meat production has led to the generation of inbred strains that show accelerated growth performance, particularly enhanced muscle growth that mostly occurs during embryogenesis [1,2]. During embryogenesis, nutrients and energy are mainly acquired from yolk, which mainly contains lipids and low levels of carbohydrates [3]. Subsequently, the health of the embryo and post-hatch chicken depends on gluconeogenesis from essential amino acids [4,5]. In recent years, researchers have found that the administration of amino acids into fertilized broiler eggs, which is called in ovo feeding, may provide poultry companies with an alternative method to increase the hatchability and muscle growth weight of newly hatched chicks [6,7]. The supplementation of nutrients into fertilized broiler eggs influences embryo development and growth during incubation and the post-hatch growth performance of chicks [7]. The nourishment and supplementation with bioactive substances such as bioactive amino acids, polyphenols, and prebiotics can enhance the immune system, decrease osteoporosis, and decrease the risk of heart diseases [8]. Similarly, previous reports have indicated that the amino acids, carbohydrates, and vitamins that are applied to eggs through in ovo feeding can improve the hatching rate, body weight, survival rate, growth performance, and marketing size [9]. Moreover, an earlier study demonstrated that the in ovo feeding site and time can affect hatchability [10].

During embryonic development, the chorioallantoic membrane develops, which can vascularize on the 12th day of the incubation period. Moreover, the embryo is surrounded by the amniotic fluid, which remains in contact with the embryonic gastrointestinal tract and enables the transport of substances from the air chamber into the intestine [11]. Several genes are associated with cellular interactions and differentiation during the organogenesis of the eye, ear, brain, skin, and tissues such as bones and cartilages; the expression of those genes is either transient or initiated during later stages of embryogenesis [12]. Some authors have indicated that the injection of amino acids into the egg on the first day is sufficient to fully support embryonic development [13,14], leading to increased hatching and breast weight [15]. It has been demonstrated that the injection of sucrose and dextrin into chicken embryos can result in a greater percentage of pectoral muscle weight than the control [5,16]. Recently, it has been reported that chicken embryos injected with L-glutamine on the first day of incubation can increase the fiber area, pectoral muscle mass, and endothelial cell proliferation while stimulating vasculogenesis and angiogenesis [17]. The in ovo administration of amino acids or peptides increases the expression levels of MyoD1 and paired box protein 7 (Pax7), which are necessary for muscle growth during embryogenesis [18].

Standardization of the injection site, needle length, and embryonic age using amino acid (Lys + Met + Cys, Thr + Gly + Ser or Ile + leu + Val) with 11-mm and 24 mm-needles on the seventh and 14th day of incubation has resulted in poor hatchability and poor muscle growth markers [7]. An in ovo injection of glutamine in conjugation with (silver nanoparticles) Ag NPs on the first day of chicken embryos increased the muscle mass [19], and L-arginine (L-Arg) in one-day-old quail embryos increased the hatchability and growth performance [20]. In our study, we checked different time intervals (eighth day, 14th day, and 18th day) and different doses of L-Ar (100, 1000, and 2500 µg/100 µL/egg) for responses related to the survival rate, hatchability, body weight, and muscle growth-related proteins such as myogenin and MyoD and immunoglobulin M (IgM) levels.

## 2. Materials and Methods

### 2.1. Ethics Statement

The experimental protocol was approved by the Institutional Animal Care and Use Committee (IACUC) of Chonbuk National University, with the project number 2017R1D1A1B03032217. Animal care and handling are in compliance with the regulations of the IAEC Guidelines for the Euthanasia of Animals: 2015 Edition. The sampling procedures complied with the “Guidelines on Ethical Treatment of Experimental Animals” (2015) No. CBNU 2015048 set by the Ministry of Science and Technology, Korea.

### 2.2. Chemicals

L-arginine, hematoxylin and eosin (H&E), and periodic acid-Schiff’s (PAS) were purchased from Sigma-Aldrich (Sigma-Aldrich, St. Louis, MO, USA). Chemiluminescent for band detection was purchased from Thermo Scientific (Rockford, IL, USA). Antibodies were purchased from ENZO Life Science (Farmingdale, NY, USA). All the laboratory glassware was acquired from Falcon Lab ware (Becton, Dickinson and Company, Franklin Lakes, NJ, USA).

### 2.3. Experimental Design and Incubation

Ross 1040 broiler chicken eggs were obtained from Samhwa-Won Jong, South Korea. On the first day of incubation, eggs were weighed (60 ± 1.36 g) and separated into different groups. Eggs were randomly divided into 13 groups (4 × 20 × 3 = replication × eggs × injection) as described in Table 1. L-Arg was injected at three concentrations (100 µg, 1000 µg, and 2500 µg/100µL/egg) on the eighth, 14th, and 18th day of the incubation period, respectively. A 0.100-mL of L-Arg (1% PBS) was injected. Immediately after the injection, the hole was sealed with liquid paraffin. Then, eggs were placed in an incubator for 20 days under standard conditions (temperature, 37.8 °C; humidity, 60%). On the 18th day, eggs were transferred to hatching boxes and promptly placed in a hatcher incubator with humidity maintained at 60% and temperature set at 37 °C to hatch chicks.

### 2.4. Survival Rate Measurement

Embryos’ survival rates during the incubation period were measured on the eighth day. Treated eggs were checked to determine the number of live and dead eggs as well as fertilized and non-fertilized ones among the total number of eggs. At 18th day of incubation, after injection, the eggs live eggs were moved to another hatching incubator with their respective experimental group. The survival rate was calculated with the following Equation (1):

(1) $$\mathrm{Survival}\;\mathrm{rate}\;\%=\frac{\mathrm{No}.\;\mathrm{of}\;\mathrm{live}\;\mathrm{eggs}}{\mathrm{No}.\;\mathrm{of}\;\mathrm{fertilized}\;\mathrm{eggs}}\;\times \;100$$

### 2.5. Hatching Rate and Body Weight Measurements

On the 21st day, hatched chicks were moved from the hatcher incubator to hatching boxes to determine hatching rates. These hatched chicks were kept without food and water at 32 °C. Then, they were weighed to record their live body weights. The hatching rate was calculated with the following Equation (2):

(2) $$\mathrm{Hatching}\;\mathrm{rate}\;\%=\frac{\mathrm{No}.\;\mathrm{of}\;\mathrm{chicks}\;\mathrm{hatched}\;\mathrm{on}\;21\mathrm{st}\;\mathrm{day}}{\mathrm{No}.\;\mathrm{of}\;\mathrm{fertilized}\;\mathrm{eggs}\;\mathrm{that}\;\mathrm{were}\;\mathrm{in}\;\mathrm{ovo}\;\mathrm{fed}}\;\times \;100$$

### 2.6. Biochemical Indices

At the end of the experimental period, the hatched chicks were sacrificed under anesthesia (diethyl ether). Blood was collected from the jugular vein in tubes for serum separation. A small amount of collected blood was immediately smeared on clean grease-free microscope slides and air-dried for micronuclei (MN) and nuclear abnormality (NA). The breast muscle and liver were collected and washed in ice-cold saline for further study. The body was cut opened; muscle and liver samples were excised, washed with ice-cold saline, and then homogenized with 0.1 M of cold phosphate buffer, pH 7.4. Concentrations of serum glutamate pyruvate transaminase (SGPT) and erum glutamate oxaloacetate transaminase (SGOT) in serum were measured using commercial kits (Asan Pharamaceuticals Co., Ltd., Seoul, Korea).

### 2.7. Micronuclei (MN) and Nuclear Abnormality (NA) Tests Using Periodic Acid Schiff’s (PAS) Staining

MN and NA were assayed in the liver by standard methods presented elsewhere [21,22]. Blood samples collected from the first day of the hatching period were immediately smeared on clean grease-free microscope slides and air dried. Afterwards, slides were fixed with methanol for 5 min at room temperature, gently rinsed with running tap water for 1 min, and immersed in a periodic acid solution for 5 min at room temperature. Then, these slides were rinsed using DH_2_O, immersed in PAS Schiff’s reagent for 15 min at room temperature, and gently washed with running tap water for 5 min. Finally, counter-staining was performed with a hematoxylin solution for 90 s. Then, slides were rinsed in running tap water for 30 s, air dried, and examined with a light microscope (100×) using immersion oil.

### 2.8. Measurement of IgM Concentration in Serum

Serum samples were collected from individual experimental animals to determine serum immunoglobulin (Ig) M levels using chicken IgM ELISA kit (Abcam, Suite B2304, Cambridge, MA 02139-1517, USA) following the manufacture’s specification. IgM levels were analyzed based on absorbance values measured at 450 nm.

### 2.9. Analysis of Heat-Shock Proteins (HSPs) by Western Blot

Proteins were extracted from 100 mg of muscle samples using radioimmunoprecipitation assay (RIPA) buffer to determine the protein expression levels of HSP-47, HSP-60, HSP-70, myoD, and myogenin in experimental groups. Protein concentrations were determined using a BIO-RAD protein assay kit (BIO-RAD). Extract samples containing 50 µg of protein were solubilized in *Laemmli buffer,* separated by 12% acrylamide gel, and then transferred to Hybond-P PVDF membranes (GE Healthcare Inc., Amersham, UK) for 60 min at 200 mA. Then, these PVDF membranes were blocked with 5% skimmed milk powder in 0.5 M of Tris-buffered saline (pH 7.4) with 0.05% Tween 20 (TBST) at room temperature for 2 h. Western immunoblotting with HSP-47, HSP-60, HSP-70, Myo-D, and myogenin are primary antibodies (1:2500 dilution) took place overnight. After washing three times with TBST, these membranes were probed with HRP-conjugated secondary antibodies (1:5000 dilutions) for 60 min at room temperature, and then washed three times with TBST (10 min each wash). Protein bands were visualized using a Chemiluminescent assay kit from Thermo Scientific for 1–5 min. Bands were imaged with an iBright™ CL1000 Imaging System (Invitrogen in Thermo Fisher Scientific, Life Technologies Korea LLC, Jeonju-si, Jeollabuk-do, Korea) and quantified using Image J Software. The relative density of the band was normalized to that of β-actin as an internal control.

### 2.10. In Silico Molecular Docking Studies

To understand the mechanism of interaction of L-Arg with heat shock protein, crystal structures of GroEL mutant A109C (PDB ID: 5OPW) [23] and human HSP70 substrate binding domain L542Y mutant (PDB ID: 5XIR) [24] were downloaded from the Protein Data Bank. Molecular docking studies were performed using the GLIDE program [25] (Version 8.5, Schrodinger LLC, New York, NY, USA). To analyze docking results and execute the protocol, the Maestro user interface (Version 8.5, Schrodinger LLC, New York, NY, USA) was employed. Validation of the protocol was performed by redocking. The structure of L-Arg was sketched using ACD/chemsketch (Freeware version). The GLIDE grid generation wizard was used to define the docking space. Docking was performed using XP (Extra Precision mode) docking protocol.

### 2.11. Histopathological Study of the Liver

Livers were collected after chickens were sacrificed, immediately fixed with 10% neutral buffered formalin (NBF), and processed in an auto processor (Excelsior ES, Thermo Scientific, Waltham, MA, USA). After embedding in paraffin, 5-μm sections were made and subjected to H&E staining. Digital images were obtained using a Leica DM2500 microscope (Leica Microsystems, Wetzlar, Germany) at fixed 100× (200×) magnification.

### 2.12. Statistical Analysis

All the values are presented as mean ± SD from 12 determinations from each group and statistically analyzed using Duncan test following ANOVAs with SAS^®^ software, version 9.4, (Institute of INC, North Carolina, USA).

## 3. Results and Discussion

### 3.1. Survival Rate and Hatchability

**Figure 1:**
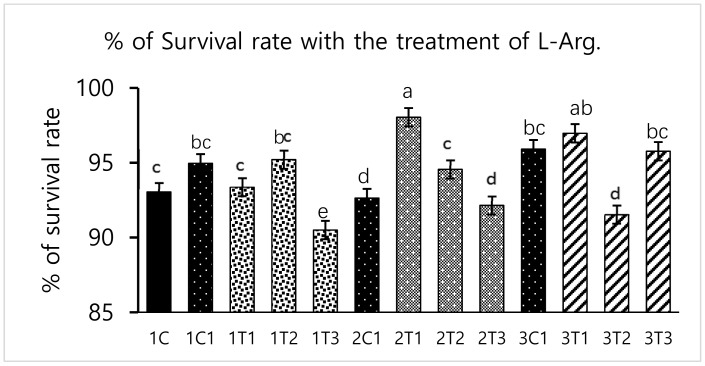
Effects of in ovo injections at different concentrations of L-arginine (L-Arg) with different developmental embryonic stages on survival rate. Small characters indicate significant differences among experimental groups at *p* < 0.01. Values are presented as mean ± SD from 12 determinations. Data were analyzed using Duncan test following ANOVAs with SAS^®^ software, version 9.4, (Institute of INC, North Carolina, USA).

Survival rate was significantly (*p* < 0.05) increased in the 2T1 and 3T1 groups than that in other groups. The lowest survival rate was observed in 1T3, 3T2, and 3T3 groups (Figure 1). These results showed that the survival rates differed depending upon the injection period and the concentration of L-Arg. Embryos may utilize in ovo administered amino acids to improve energy status and save muscle protein to improve their enteric development, hatching, and survival rate [26]. In our study, the same mechanism might have occurred; the administration of L-Arg could improve the survival rate at the minimal concentration (3T1) on the 14th day of the injection period (Figure 1). However, during incubation, an excess of amino acids such as glycine and proline failed to improve embryo development [27]. The same attributes could have been observed in our current study: that a maximum concentration of L-Arg affects the embryonic growth.

**Figure 2:**
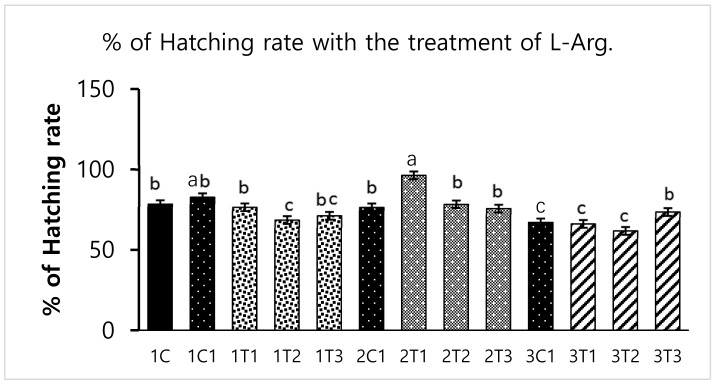
Effects of in ovo injections at different concentrations of L-Arg with different developmental embryonic stages on hatching rates. Small characters indicate significant differences among experimental groups at *p* < 0.01. Values are presented as mean ± SD from 12 determinations. Data were analyzed using Duncan test following ANOVAs with SAS^®^ software, version 9.4, (Institute of INC, North Carolina, USA).

**Figure 3:**
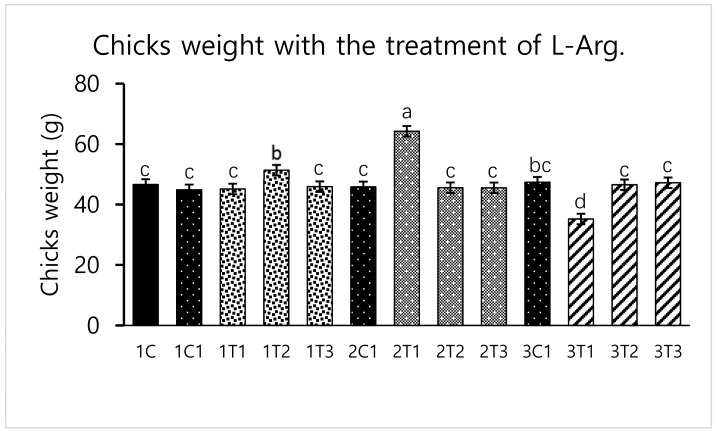
Effects of in ovo injections at different concentrations of L-Arg with different developmental embryonic stages on Chicks weight. Small characters indicate significant differences among experimental groups at *p* < 0.01. Values are presented as mean ± SD from 12 determinations. Data were analyzed using Duncan test following ANOVAs with SAS^®^ software, version 9.4, (Institute of INC, North Carolina, USA).

Different concentrations of L-Arg injected in embryos can influence biological molecules and toxicity during embryogenesis. However, several studies have reported that higher doses of L-Arg become toxic, which can cause significantly increased mortality rates and impaired weight gain, whereas chicks injected with lower concentrations of L-Arg (1.0%) showed better growth performance than those injected with a higher concentration (1.5%) of L-Arg [28]. In a parallel effect revealed in our current study to a lower concentration of L-Arg (2T1) on the 14th day of injection increasing the hatching rate (96.29%), body weight (64.25 g) also increased the survival rate (98.03%) compared to other groups (Figure 1, Figure 2 and Figure 3). The in ovo injection of L-Arg to late-term embryos can increase the body weight (5% to 6%) compared to controls [5]. In addition, in ovo administration of all 20 different amino acids can increase chick weight by 3.6% and 2.1%, respectively [10]. The in ovo administration of amino acids might have stimulated the utilization of amino acids with a concomitant decrease in the degradation of amino acids by the embryo [29]. In ovo feeding of L-Arg resulted in higher embryo weight due to an increase in muscle mass [30]. The in ovo injection of L-Arg could be utilized by the embryo, resulting in increased muscle mass and a heavy embryo, which can increase the hatching rate [31]. L-Arg may attenuate adverse effects of rearing chickens under cold ambient temperatures or at high altitudes [32]. Furthermore, feeding broiler chickens with a diet that is deficient in L-Arg under cold stress at high altitudes can depress nitric oxide synthesis, decrease feed intake, reduce body weight gain, and increase the right ventricle to total ventricle weight ratio, mortality rate, and ascites mortality [31]. A previous study reported that a lower percentage (1.36%) of L-Arg supplemented to broiler eggs was more easily digestible than a higher percentage of arginine, and it could obtain the highest egg weight [33]. Albeit, a low dose of L-Arg stimulates the secretion of the growth hormone, which could increase the body weight [21]. The same mechanism might occur in our present study; L-Arg appeared to improve the body weight of chicks in group 2T1 (Figure 3). However, the body weights did not significantly vary among the other groups. Hatchability, gut microflora population, immune-related gene expression, and muscle fiber increased as a result of the 12–14-days in ovo injection of various substances such as Raffinose, *Lactococcus lactis*, and *Silybum marianum* extract [34,35,36]. Hence, a low dose (2T1) of L-Arg injected on the 14th day of the incubation period could improve body weight; the reason behind this might be that the lower concentration of L-Arg may stimulate the growth hormones in the middle stages of embryonic development when compared to the early (eighth day) and late (18th day) embryonic stages.

### 3.2. Biochemical Indices (SGOT and SGPT)

**Figure 4:**
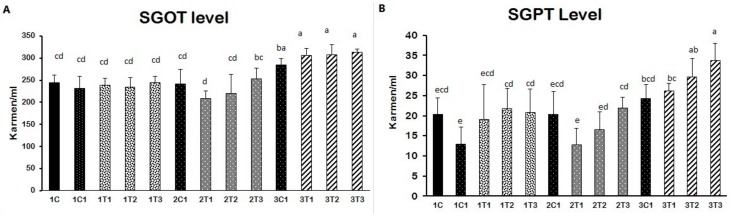
(**A**,**B**) Effect of in ovo feeding on broiler eggs with different concentrations of L-Arg at different developmental embryonic stages and effects on serum glutamate oxaloacetate transaminase (SGOT) and serum glutamate pyruvate transaminase (SGPT) concentrations in serum. Small characters indicate significant differences among the experimental groups at *p* < 0.01. Values are presented as mean ± SD from 12 determinations. Data were analyzed using Duncan test following ANOVAs with SAS^®^ software, version 9.4, (Institute of INC, North Carolina, USA).

Elevated SGOT and SGPT levels indicate improper liver function due to damages of the cell integrity and cell membrane in the liver. Our results revealed that the injection of L-Arg at all doses except the lower dose affected SGOT and SGPT levels on the eighth and 18th-day embryonic stages (Figure 6A). SGOT and SGPT levels were significantly decreased in the 2T1 and 2T2 groups of embryos compared to 2T3, 3C1, 3T1, 3T2 and 3T3. Increased levels of SGOT and SGPT in the blood are conducive to liver function damage [37,38,39]. In fact, free radicals can attack hepatocytes and release stored SGPT to re-enter the blood serum [40]. A lower concentration of L-Arg supplementation caused a greater percentage reduction in SGOT and SGPT levels in sickle cell anemia subjects [39]. The supplementation of L-Arg to mice in higher concentrations showed that increased SGOT and SGPT levels had been linked to damage to hepatic cells and hemolysis [41]. The cause of liver damage is unclear. Hence, confirming that a higher concentration of L-Arg might have damaged the hepatic cell through the elevation of SGOT and SGPT in the 1T1, 1T2, 1T3, 3T1, 3T2 and 3T3 groups. On the other hand, the 2T1 group of injected chicken embryos could re-back the SGOT and SGPT levels compared to the other groups. Stimulating the action of nitric oxide (NO) production by L-Arg results showed that it improved the degree of the hepatocellular structure by blocking of B-cell lymphoma-2 (Bcl-2) and tumor necrosis factor-α (TNF-α) [42]. In addition, L-Arg at 1 g/day decreased the liver enzymes such as SGOT and SGPT through increasing the nitric oxide (NO) synthesis. NO synthase plays an important role in liver injury through inducible nitric oxide synthase (iNOS) pathways [43]. The same mechanism could be involved in our current study, too. This same mechanism that might have occurred in our study could be that the production of NO reduces necrosis and apoptosis by attenuation of the inflammatory pathway, which in turned prevented the hepatotoxicity. Moreover, it also improved the hepatobiliary function, and the ultrastructure of liver results reduced the SGOT and SGPT levels in L-Arg treatment in the lower dose (2T1) on the 14th day injection of embryos (Figure 4).

### 3.3. Micronuclei (MN) and Nuclear Abnormality (NA) Tests Using Periodic Acid-Schiff’s (PAS) Staining

**Figure 5:**
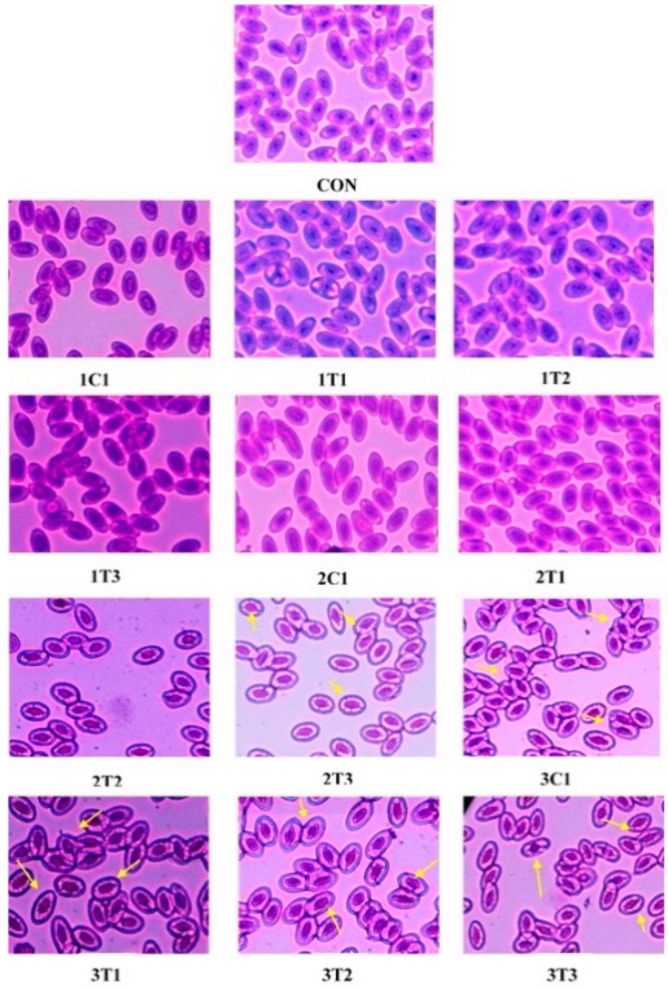
Photomicrographs of erythrocytes with normal nuclei in the peripheral blood cells of experimental groups. Micronuclei and nuclear abnormalities such as blebbed nuclei or lobed nuclei are indicated by an arrow (“→”).

The wide use of different doses of L-Arg at three different incubation periods requires examining the genotoxic activity in peripheral blood by the method of [44]. MN and NA tests were conducted to examine peripheral blood cells in all the groups of experimental chicks (Figure 5). The MN test can measure subcellular processes of chromosomal breaks (clastogenesis) or cell spindle malfunctions (aneugenesis) as well as the formation of mitochondrial disruption and nuclear DNA, which can lead to mitochondria-dependent apoptosis in chicken embryos as an indicator of chromosomal damage [45]. Similar results were obtained in our current experiment: the MN and NA in peripheral blood erythrocytes were observed, which clearly demonstrates the higher genotoxicity of a high dose of L-Arg on the eighth, 14th, and 18th day of the incubation period. Moreover, the 2T1 and 2T2 groups showed a normal architecture of nuclei in peripheral blood cells, which was similar to the control group. Figure 5 shows marked inflammation around the periportal region with microvesicular and macrovesicular fatty infiltration (yellow arrows).

### 3.4. Protein Analysis by Western Blot

**Figure 6:**
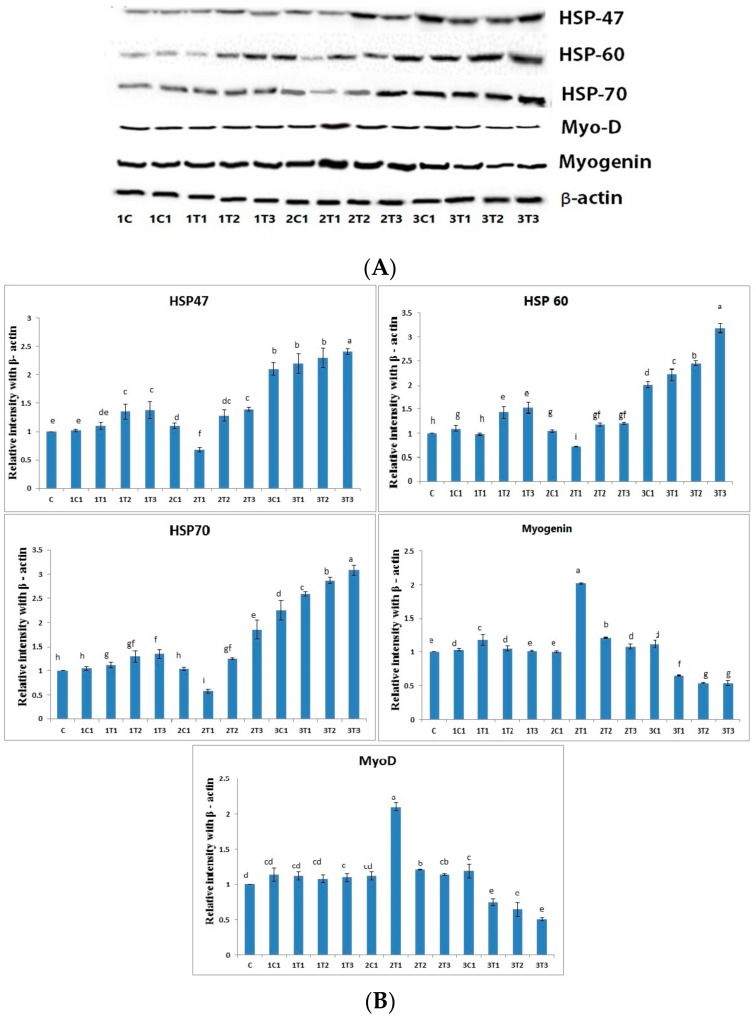
(**A**) Effects of expression levels of L-Arg, heat shock protein (HSP)-47, HSP-60, and HSP-70 as well as myogenin and myoblast determination (MyoD) protein expressions in different stages of chicken embryos at different doses. Small characters indicate significant differences among experimental groups at *p* < 0.01. (**B**) The bar graph represents the quantitative expression of different proteins in all the groups. Data are expressed as the ratio of relative intensity with β-actin. Values are presented as mean ± SD from 12 determinations.

Western blot was performed in muscles to determine whether the different doses of L-Arg supplemented at various days of the incubation period may alter the protein levels of the HSP family such as HSP-47, HSP-60, and HSP-70. As shown in Figure 6, the protein expressions of HSP-47, HSP-60, and HSP-70 were significantly (*p* < 0.01) down-regulated in the 2T1 group compared to other groups. Moreover, their levels in the 3T2 and 3T3 groups were significantly (0.01) up-regulated compared to the 2T1 and 2T2 groups, although the protein expressions of HSP-60 and HSP-70 showed no significant difference (*p* < 0.01) among the 1T1, 1T2, 1T3, and 2T1 groups. Moreover, HSP-46, HSP-60, and HSP-70 were down-regulated in the 2T1 group compared to those in the other groups, whereas there was no significant difference in their levels between 1C and 2T1. HSP-70 is a reliable index of stress in chickens, while “3-hydroxyl-3-methyl-glutaryl coenzyme A reductase” has been used as an indicator of stress [46]. Pretreatment with L-Arg markedly reduced the dramatic down-regulation of HSP-60 and HSP-70 in hypoxic rat model. The increased expression of HSP-60 and HSP-70 might be related to their leakages from tissue, which can cause tissue injury due to free radical production [47,48]. Tissue injury might be caused by nitric oxide, a free radical, through the stimulation of endothelial cells and neutrophils that is generated from a higher dose of L-Arg [49]. Hence, the present results may suggest that the increased levels of HSP-47, HSP-60, and HSP70 in high doses of L-Arg may have a major role in tissue injury. The results of the study show that the increase of HSP-60 and 70 may be involved in tissue injury in the 3T1, 3T2, and 3T3 groups due to free radical production. The 2T1 group can prevent tissue injury via the down-regulation of HSP-46, HSP-60, and HSP-70. Moreover, the protein expressions of myogenin and MyoD were significantly up-regulated in the 2T1 group, whereas they were down-regulated in the 3T1, 3T2, and 3T3 groups compared to the other experimental groups.

Oxidative stress can cause muscle atrophy by reducing myogenic differentiation markers such as myogenin and MyoD in skeletal muscles [50]. Some growth factors, namely cytokines and oncogenes, suppress the activity of myogenin and MyoD, thus resulting in decreased in the mass of muscle, which is defined as muscle atrophy [51]. A previous study reported that myogenic regulatory factors—mainly MyoD and MRF4—are only expressed later in different embryonic muscle groups as a result of increased muscle mass [52]. L-Arg increased the muscle cell as well as myogenin and MyoD under oxidative stress. Moreover, results from a previous experiment demonstrated that lower doses of L-Arg could promote HSP70 expression in pig intestine [53]. Nevertheless, our present study has proved that increasing the concentration of L-Arg on the eighth and 18th day of the injection period could up-regulate the expression of HSP-60 and HSP-70; this effect might be through whey protein hydrolysate, which indicates the improper use of a functional food ingredient. Moreover, the L-Arg on the 14th day with (100 μg/100 μL/egg) promoted the muscle mass through the up-regulation of MyoD and myogenin due to their free radical scavenging activity.

### 3.5. Measurement of IgM Concentration in Serum

**Figure 7:**
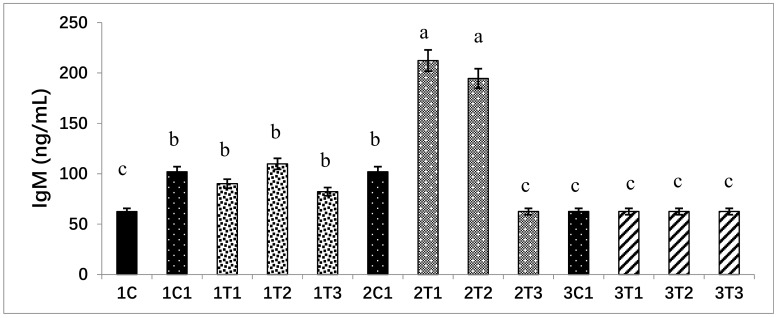
L-Arg induces immunoglobulin M (IgM) levels in different stages of chicken embryos at different doses. Small characters indicate significant differences among experimental groups at *p* < 0.01. Values are presented as mean ± SD from 12 determinations. Data were analyzed using Duncan test following ANOVAs with SAS^®^ software, Version 9.4, (Institute of INC, North Carolina, USA).

Concentrations of immune response markers such as IgM in all the experimental groups were analyzed. The duration and amount of L-Arg supplementation may influence immune status. Short-term supplementary L-Arg can influence the immunity power, because L-Arg has antioxidant and anti-inflammatory effects [54,55]. It can attenuate inflammatory reactions by suppressing the generation of inflammatory mediators such as inflammatory cytokines and C-reactive protein, which play major roles in the progression of tissue damage and organ dysfunction [56]. The treatment of L-Arg shows improved renal function through improved immune function [57]. Levels of IgM could provide an overall picture of immune function. It has been recently demonstrated that L-Arg can increase the specific immune response against infectious bursal disease (IBD) in chickens [58]. L-Arg provided by treatment has been reported to be the sole precursor of nitric oxide with lots of immune functions and growth performance [59]. These same biological attributes might be present after a low dose of L-Arg injection on the 14th day of the incubation period. It may improve immunity via the generation of IgM and the suppression of inflammatory cytokines and C-reactive protein (Figure 7).

### 3.6. Histopathology (H&E) Staining

**Figure 8:**
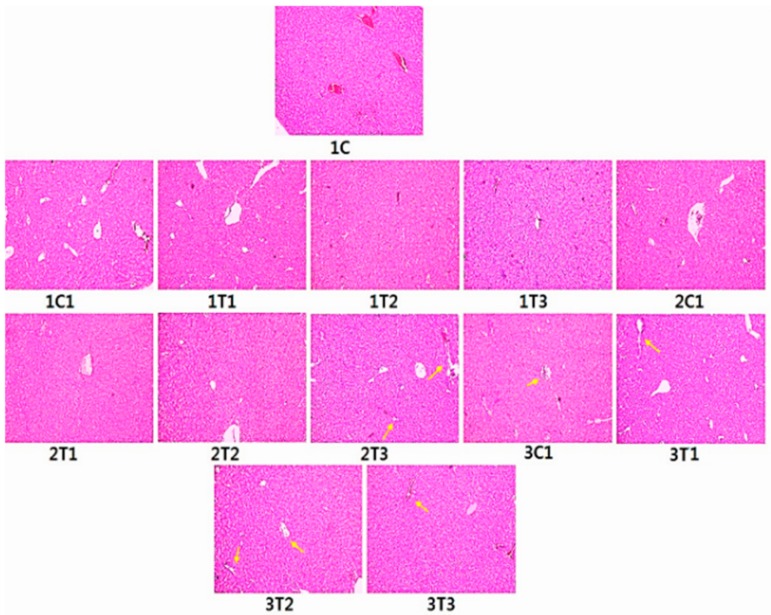
Histopathology of liver using hematoxylin and eosin straining. Sections from control chicks’ hepatic lobule indicate complete structures. The liver cell has a regular shape that is within normal limits. Intracytoplasmic vacuoles are shown in hepatocytes around the centrilobular region in the 1T3, 2T3, 3T1, 3T2, and 3T3 groups. Hepatocyte marked inflammation around the periportal region, with microvesicular and macrovesicular fatty infiltration (yellow arrows) liver cells appearing near necrosis with nuclear fragmentation in the 3T2 and 3T3 groups. The hepatocyte gap was also increased in the 2T3 3T1, 3T2, and 3T3 groups. There was no difference between the control and the 2T1 group.

Figure 8 shows the histology of liver of all the experimental groups. Sections from the control group exhibited a complete structure and regular shape of liver cells. Sections from the 1T1 and 2T1 groups showed normal hepatocyte gap compared to the 1C and 1C1 groups. Sections from the 1T3, 2T3, 3T1, 3T2, and 3T3 groups appeared with intracytoplasmic vacuoles in hepatocytes around the centrilobular regions. Moreover, hepatocyte tubes were surrounded by inflammatory cells and showed necrosis with nuclear fragmentation in the 3T2 and 3T3 groups. The hepatocyte gap was increased in the 2T3, 3T1, 3T2, and 3T3 groups. The hepatocyte gap appeared in normal architecture in the 1C, 1C1, 2C1, 3C1, 1T1, and 2T2 groups. The degeneration of livers was observed for birds when treated with 167 and 334 mg/L of L-Arg, which had an adverse effects on organs [60]. The liver after treatment with L-Arg (334 mg/L) had congested vascular spaces and periportal mononuclear inflammatory infiltration [61]. The addition of L-Arg to poultry diets is required to avoid harmful influences of excessive free radicals produced during normal metabolism [62]. Dietary L-Arg supplementation plays a key role in enhancing meat quality. Increased L-Arg and betaine supplementation alleviates total body fat deposition and fatty liver [63,64]. Additionally, supplementation with high doses (50% and 100%) of L-Arg has negative effects on the structure of the liver of Sasso birds proved by H&E staining. However, our current results showed that in ovo injection with low doses (2T1) of L-Arg on the 14th day of egg embryo development did not have any negative effects compared to higher doses of L-Arg on the eighth or 18th day of the incubation period.

### 3.7. In Silico Molecular Docking Studies

**Figure 9:**
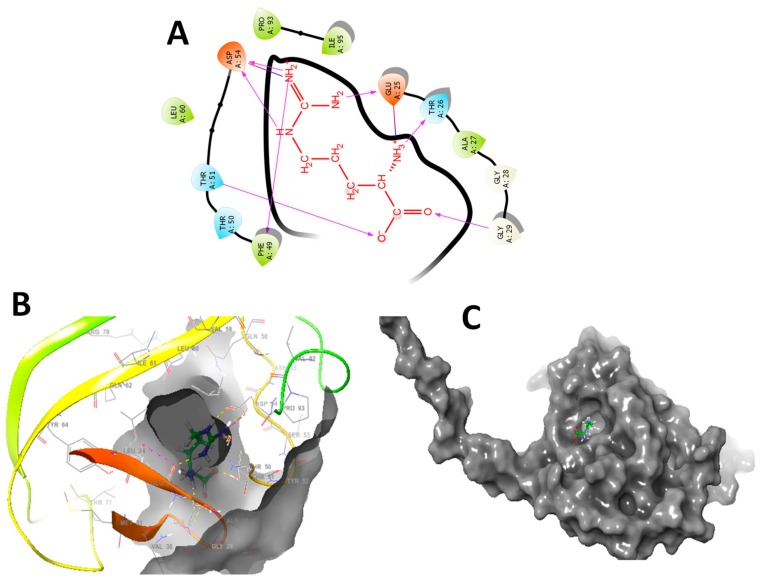
(**A**) 2D docking interaction of L-arginine with the active site of 5XIR. (**B**) 3D docking of L-arginine in the active site of 5XIR. (**C**) Docking packing representation of L-arginine with suitable binding pockets of 5XIR.

The in silico molecular docking of L-Arg was studied. The entire glide, E model scores, and hydrogen bond interactions are presented in Figure 9. The main aim of the molecular modeling study is to understand the interactions of functional groups present in L-Arg with residues of targeted proteins theoretically. Heat shock protein 70 (HSP70) is one of the main nonglobin proteins, which has a similar structure in almost all living organisms. Between organisms as varied as yeast, chicken, Drosophila, and human, HSP70 is highly conserved. A previous study has described that HSPs are antigenically linked to the chicken HSPs by means of rabbit polyclonal antibodies [25]. In the same way, the similarity of HSPs extends to a DNA sequence. The chicken and human HSP70 genes are 64–72% similar (homologous) in the obtained amino acid sequences [65]. In addition, the HSP70 gene(s) of chicken, Drosophila, mouse, and human are very much conserved with the comparison of the DNA sequence [66,67]. Therefore, in the present study, solution structure for the human HSP70 substrate binding domain L542Y mutant (PDB id: 5XIR) was selected for the in silico molecular modeling study with L-arginine [68].

As presented in Figure 9, the main aim of the molecular modelling study is to understand interactions of functional groups present in L-Arg with residues of targeted proteins theoretically. In L-Arg, primary and secondary amines have strong hydrogen bonding interactions with protein residues. In brief, the molecular interaction of L-Arg with 5XIR protein and primary and secondary amines established a strong affinity with protein resides such as ASP A 328, ASP A 83, and HIS A 401. In addition, the carboxyl group present in the molecule also exhibits good hydrogen bonding interactions with LYS A 498. In a similar way, L-arginine with 5XIR protein showed good molecular interactions with ASP A 54, PHE A 49, GLU A 25, THR A 26 and GLY A 29 (Figure 9A).

**Figure 10:**
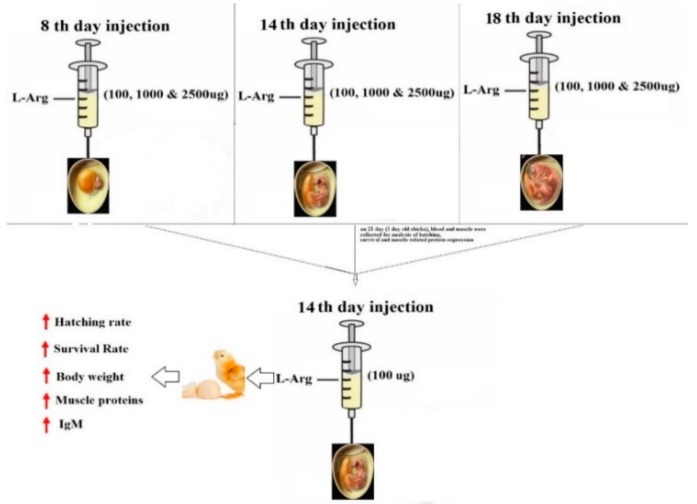
Possible mechanism of high doses and low doses of L-Arg on toxicity and muscle growth in chicken embryo.

The molecular modelling study revealed that L-Arg was bound onto similar active site cavity in the protein molecule. Superposition of interactions of an active site of L-Arg and amino acid residues of 5XIR protein is evidently portrayed in Figure 9B,C. The molecular docking of L-Arg with 5XIR protein is exposed to seven hydrogen bonding interactions, respectively with corresponding active site of the protein molecule. Besides, L-Arg showed strong hydrogen bonding interactions with good surface molecular interactions due to the presence of primary, secondary amines, and carboxylic acid groups. These relationships among 5XIR protein, and L-Arg might explain the experimental activity of them. Further research is going on in a due course to explore their possible modes of action. It is possible that L-Arg might block the activity of HSPs, and activation of Myogenin and Myo-D as well improved the immunoglobulin levels, thereby regulating the muscle growth (Figure 10).

## 4. Conclusions

In this study, we described a suitable embryonic developmental stage for the accessibility of in ovo injection using L-Arg at different concentrations for the first time. The injection of L-Arg on the 14th day at 100 μg/μL/egg enhanced both hatching and survival rates. It also increased body weight and immune response (IgM). In addition to oxidative stress, a sign of genotoxic effect was also observed in peripheral blood cells in which a presence of micronuclei and nuclear abnormalities such as blebbed nuclei, lobed nuclei, and notched nuclei were observed in the 2T3, 3T1, 3T2, and 3T3 groups. Histology from the control and 2T1 groups showed normal architecture, while injection on the 18th day of incubation and first day of chicks showed liver tissue damage. Overall results demonstrate that the optimum dose is 100 μg/μL/egg, and the optimum injection stage is on the 14th day to improve the immunity, hatching, and survival rate, which can be used for the poultry industry. In ovo injection in early and late embryonic stages could not offer good benefits for survival, hatching rate, or muscle development. If we choose the middle stage of embryonic development for in ovo injection, L-Arg might be able to promote muscle growth and improve the immune power without inducing adverse effects on the liver.

## Figures and Tables

**Table 1:** Experimental design for dose (L-Arg) and embronic stage (eighth day, 14th day, and 18th day) fixation.

Group	Dosage	No. of Eggs & No. of Replication	Total No. of Eggs
1C	Control	20 eggs × 4 replicates	80
1C1 (8th day)	PBS/100 µL/egg	20 eggs × 4 replicates	80
1T1 (8th day)	100 µg/100 µL/egg	20 eggs × 4 replicates	80
1T2 (8th day)	1000 µg/100 µL/egg	20 eggs × 4 replicates	80
1T3 (8th day)	2500 µg/100 µL/egg	20 eggs × 4 replicates	80
2C1 (14th day)	PBS/100 µL/egg	20 eggs × 4 replicates	80
2T1 (14th day)	100 µg/100 µL/egg	20 eggs × 4 replicates	80
2T2 (14th day)	1000 µg/100 µL/egg	20 eggs × 4 replicates	80
2T3 (14th day)	2500 µg/100 µL/egg	20 eggs × 4 replicates	80
3C1 (18th day)	PBS/100 µL/egg	20 eggs × 4 replicates	80
3T1 (18th day)	100 µg/100 µL/egg	20 eggs × 4 replicates	80
3T2 (18th day)	1000 µg/100 µL/egg	20 eggs × 4 replicates	80
3T2 (18th day)	2500 µg/100 µL/egg	20 eggs × 4 replicates	80

Note: In Ovo Injection and Treatment Groups.
